# Individual and systemic variables associated with prolonged grief and other emotional distress in bereaved children

**DOI:** 10.1371/journal.pone.0302725

**Published:** 2024-04-30

**Authors:** Paul A. Boelen, Mariken Spuij

**Affiliations:** 1 Department of Clinical Psychology, Utrecht University, Utrecht, The Netherlands; 2 ARQ National Psychotrauma Centre, Diemen, The Netherlands; 3 Department of Child and Adolescent Studies, Utrecht University, Utrecht, The Netherlands; 4 TOPP-zorg, Driebergen-Rijsenburg, The Netherlands; Ariel University, ISRAEL

## Abstract

Most children confronted with the death of a loved one do not experience persisting psychological problems. However, for some, acute grief reactions develop into prolonged grief disorder (PGD) and other mental health problems. Research findings suggest that bereavement outcomes in children are associated with negative cognitions and avoidant coping and with different parenting behaviours. However, knowledge about factors influencing grief in children is still limited and few studies have examined the relative impact of psychological (individual-level) variables and systemic (family-level) variables in affecting their responses to loss. The aim of the current study was to examine the association of different bereavement outcomes in 8–18 year old children (including levels of self-rated PGD, depression, and posttraumatic stress (PTS)) with sociodemographic variables, individual-level variables (including negative cognitions and anxious and depressive avoidance), and family-level variables (including the severity of caregiver’s PGD, depression, and anxiety, and indices of parenting behaviours, rated both by children and by their caregivers). Questionnaire data were used from 159 children plus one of their caregivers, gathered as part of the pre-treatment assessment in a randomized controlled trial. Results showed that most of the children’s bereavement outcomes, including PGD severity and PTS severity, were associated with indices of negative cognitions and avoidance behaviours. Caregiver’s depression and anxiety showed a very small, yet significant, association with two children’s outcomes. Caregiver-rated reasoning/induction (one index of parenting behaviours) showed a small association with children’s PTS-related functional impairment. Exploratory analyses indicated that the linkage between parenting behaviour and children’s outcomes may be moderated by whether the behaviour comes from father or mother. This is one of the first studies examining how individual cognitive behavioural variables plus the mental health of caregivers and indices of parenting may affect PGD and other outcomes in bereaved children. The findings provide tentative indications that individual and family-level variables influence these outcomes, albeit that more research is urgently needed.

## Introduction

Confrontation with the death of a parent, sibling, or other relative is one of the most frequent and impactful life-events that children may experience. Like adults, most children do not experience persisting psychological problems following loss [1, 2]. Yet, for some, acute grief reactions develop into persistently distressing and disabling grief reactions or other mental health problems. Recently, the Diagnostic and Statistical Manual of Mental Disorders, Fifth Edition, Text Revision [DSM-5-TR; 3] and the Eleventh Revision of the International Statistical Classification of Diseases and Related Health Problems [ICD-11; 4] included prolonged grief disorder (PGD) as a novel disorder, encompassing persistent separation distress and accompanying symptoms (e.g., anger, disbelief) present to a distressing and disabling agree, at least six months after the loss (12 months for adult PGD as defined in DSM-5-TR).

Considering increasing recognition that in adults and children alike, not all grief is healthy, there is a need to understand why some children develop pervasive grief whereas others recover easily following a period of transitory grief. There is still a scarcity of knowledge about factors affecting grief in children. Regarding sociodemographic factors, it is uncertain whether boys and girls have a different risk of unhealthy grief [5]. As for situational variables, studies have found that loss of close others is associated with more intense grief compared to losing more remotely related relatives [6]. Research findings on cause of death are inconclusive with some [7] but not other [8] studies showing that confrontation with unnatural loss renders children more vulnerable to persistent grief [see also 9]. With respect to psychological processes mediating bereavement outcomes, there is evidence that persistent grief is associated with negative cognitions [10] and maladaptive coping behaviours [5]. Despite this research, knowledge about factors influencing grief in children is still limited. In addition, methodological differences between studies in terms of samples and measurements of unhealthy grief make it difficult to assess the relative importance of the different factors studied.

Notable too is that knowledge on the role of systemic variables that may affect the mental health of bereaved children is still scarce [9, 11]. Two relevant variables in this regard are caregiver’s mental health and the caregiver’s parenting behaviours [12]. The death of a parent or child within a family elicits grief and mourning in caregivers that may influence the grief of the remaining children, both directly and indirectly. For instance, witnessing the caregiver’s pain or mimicking his/her unhelpful coping strategies may strengthen the children’s grief directly. Moreover, the pain of caregivers may interfere with their responsiveness to the children’s needs, thereby indirectly increasing the children’s mental health issues. With respect to parenting behaviours, it is conceivable that positive parenting (e.g., providing support and involvement and granting autonomy) mitigates children’s grief, whereas a lack of positive parenting exacerbates this grief. Indeed, work from Sandler and colleagues [e.g., 13] has shown that increased positive parenting was associated with lower children’s mental health [14, see also 11]. Moreover, interventions increasing positive parenting successfully reduced bereaved children’s mental health problems [15].

These earlier studies are important but some of them are limited in that they focused on parentally bereaved children only and did not examine PGD symptoms. In the neighbouring field of traumatic stress studies, a growing literature has examined caregiver’s mental health and caregiver’s parenting behaviours in children’s posttraumatic stress (PTS) following traumatic events. Research has confirmed that parental and children’s PTS after a child’s exposure to a traumatic event are associated, with associations being moderated by factors such as trauma type and the method used to assess PTS [16]. Evidence also confirms that children’s PTS is associated with (increased) positive and (decreased) negative parenting, albeit that associations are generally small [17]. These findings tell us something about how caregiver’s mental health and parenting behaviours may affect children’s responses to adverse events. However, considering that traumatic events and loss events can have a very different impact on the family system, these findings cannot simply be generalized to consequences of childhood bereavement.

There is still a need to advance our understanding about factors accounting for the variability in children’s emotional distress following the death of a parent, sibling, or other close person. It seems particularly important to further our knowledge about the relative impact of psychological (individual-level) variables and systemic (family-level) variables in affecting children’s responses to loss. This knowledge bears theoretical relevance to our understanding of factors affecting children’s grief and may inform the refinement of treatment interventions to mitigate this grief.

Accordingly, the overarching aim of this study was to examine the associations of different bereavement outcomes in children (including levels of self-rated PGD, depression, PTS, and functional impairment, and caregiver-rated internalizing and externalizing) with sociodemographic, loss-related, individual-level, and family-level variables. In so doing, we used data from children and one of their caregivers participating in a randomized controlled trial (RCT) examining the effects of cognitive behavioural therapy (CBT) for PGD [18]. Two theoretical frameworks guided this research. The first was the cognitive behavioural conceptualization of prolonged grief [PG; 19]; this approach postulates that acute grief may develop into PG and associated symptoms under the influence of, e.g., negative cognitions (about the self, life, the future) and maladaptive avoidance strategies (including depressive avoidance of activities that could foster adjustment and anxious avoidance of stimuli reminding of the loss). The second was the theoretical approach underpinning Sandler et al.’s family bereavement program [12, 13] proposing that psychological functioning of caregivers, plus aspects of positive parenting (including warmth and involvement, reasoning and induction, and autonomy-granting) significantly impact children’s adjustment to bereavement.

The aims of this study were fourfold. First, we examined the associations of children’s bereavement outcomes with three individual-level variables, namely children’s negative cognitions and anxious and depressive avoidance behaviours.

Second, we examined the associations of children’s bereavement outcomes with the severity of caregiver’s symptom-levels of PGD, depression, and anxiety.

Third, we examined the associations of children’s outcomes with parenting behaviours, specifically warmth and involvement, reasoning and induction, and autonomy-granting, both as perceived by the child as well as from the perspective of the caregiver.

Last, we considered all individual-level and family-level variables significantly correlated with one or more of the children’s bereavement outcomes together, to examine which of these were most important correlates of these outcomes. We anticipated that both individual-level and family-level variables would be associated with PGD and some of the other indices of children’s mental health. Given the lack of research in this area, we had no specific hypotheses about the relative importance of the variables in explaining variance in children’s mental health outcomes.

## Method

### Participants and procedure

Participants were all recruited from eight outpatient clinics in the Netherlands (from 2010–2015) for a study evaluating CBT for PGD [18, 20]. They were all self-referred or referred by local professionals. Following regular screening at the outpatient clinic, eligible children and their caregivers (i.e., biological, step, or foster parents) were informed about the study, using written information and verbal explanation. To be included, children had to be aged between 8 and 18 years, to be confronted with the loss of a close person (i.e., (grant)parent, sibling, friend, someone else considered close), and to be experiencing pervasive PGD symptoms as primary complaint and reason for seeking help. Reasons for exclusion were mental disability, severe conduct or developmental disorders, undergoing concurrent psychological treatment (other than the treatments evaluated in the RCT) or pharmacological treatment, reporting substance abuse or dependence, psychotic symptoms, severe depression, and suicidality. Written informed consent was obtained from caretakers and—in keeping with ethical regulations—verbal assent was obtained from children <11 years and written consent from children ≥12 years. Next, pre-treatment measures were administered. Children completed those in the presence of a therapist. Caregivers completed these on their own. Ethical approval for the study procedures was obtained from an independent medical ethics committee (CCMO number NL30528.041.09). In total, 161 children completed the pre-treatment assessment. Data of this group were analysed except for data from two children, for whom no measures completed by caregivers were available. Authors had access to information that could identify individual participants during data collection, because they were responsible for storage of the data in accord with ethical regulations. Anonymized data were stored on a secured server, using pass-word protection. **Table 1** summarizes characteristics of the sample for the current study (N = 159). (Note that in our RCT, 134 participants were included. Of all 161 children completing the pre-treatment assessment, 27 were excluded because PGD-symptoms were not the primary problem and reason to seek therapy in that study [18]. As described in the manuscript on our RCT [18], these 134 participants were randomly allocated to a condition with CBT (called CBT Grief-Help) or supportive counselling.

**Table 1:** Demographic characteristics, loss-related characteristics, and scores on children’s bereavement outcomes (N = 159).

***Demographic Characteristics***	
Sex (*N* (%))	
Male	79 (49.7)
Female	80 (50.3)
Age (*M* (*SD*))^a^	13.13 (2.81)
***Loss-Related Characteristics***	
Deceased is (*N* (%))	
Parent	109 (68.6)
Sibling	9 (5.7)
Other close person	41 (25.8)
Cause of death is (*N* (%))	
Illness	94 (59.1)
Unnatural (accident, suicide, homicide)	31 (19.5)
Sudden medical cause (e.g., heart attack)	28 (17.6)
Unknown	6 (3.8)
Death was expected/anticipated by participants? ^a^	
Yes	55 (34.6)
No	102 (64.2)
Time since loss in months (*M* (*SD*))	40.02 (37.15)
***Symptom Scores***	
IPG-C (*M* (*SD*), range)	54.79 (11.59)
CPSS (CPSS) (*M* (*SD*), range)	15.77 (9.59)
Functional impairment items CPSS (*M* (*SD*), range)	2.29 (1.80)
CDI (*M* (*SD*), range)	12.95 (7.50)
CBCL Internalizing (*M* (*SD*), range)	14.35 (8.85)
CBCL Externalizing (*M* (*SD*), range)	11.86 (8.60)

Note. There were occasional missing values for some variables. CBCL = Child Behavior Checklist.

CDI = Children’s Depression Inventory. CPSS = Child Posttraumatic Stress Disorder Symptoms Scale. IPG-C = Inventory of Prolonged Grief for Children.

^a^ There were 2 missing values for this variable.

### Measures

#### Children’s PGD symptoms

The Inventory of Prolonged Grief for Children [IPG-C; 21] was used to measure children’s PGD symptoms. The IPG-C is a 30-item children/adolescent version of the Inventory of Complicated Grief Revised (ICG-R), an adult measure developed by Prigerson and Jacobs [22]. It measures PGD symptoms—as now included in DSM-5-TR—and other markers of unhealthy grief (e.g., “I cannot believe that s/he died”). Respondents rate symptom frequency in the last month on three-point scales (1 = *almost never*, 2 = *sometimes*, 3 = *always*). Cronbach’s alpha in this sample was .91.

#### Children’s PTS symptoms

PTS symptoms were assessed with the Child Posttraumatic Stress Disorder Symptoms Scale [CPSS; 23]. The CPSS includes 17 symptoms of posttraumatic stress-disorder as defined in DSM-IV [24]. Children rated the occurrence of symptoms during the preceding two weeks, with the loss as the anchor-event (e.g., “Having upsetting thoughts or images about the loss that came into your head when you didn’t want them to”) on four-point scales (0 = *not at all/only once a week* through 3 = *almost always/five or more times a week*). The scale’s alpha was .87.

#### Children’s functional impairment associated with posttraumatic stress (PTS)

Children’s functional impairment associated with PTS was assessed using seven items that are part of the CPSS [23]. These items ask children to rate whether the PTS led to problems in seven domains (e.g., “Doing you chores”, “Schoolwork”) on a dichotomous yes/no scale. Both the CPSS and its functional impairment items have good reliability and validity [23, 25]. Cronbach’s alpha of the functional impairment items was .66.

#### Children’s depression symptoms

Depressive symptoms were assessed using the Children’s Depression Inventory [CDI; 26]. The CDI contains 27 groups of three statements representing depressive symptoms at increasing levels of severity (“I have fun in many things”; “I have fun in some things”; “Nothing is fun at all”), scored from 0 = *symptom absent* through 2 = *symptom present always/most of the time*. Respondents select statements best describing their state in the preceding week. English [cf. 27] and Dutch versions [28] have adequate psychometric properties. In the present sample, the Cronbach’s alpha was .87.

#### Children’s grief cognitions

Children’s negative cognitions connected with their loss were assessed using the 20-item Grief Cognitions Questionnaire for Children (GCQ-C). This measure was developed by Spuij et al. [10] based on the adult version of the measure [29]. The GCQ-C includes 20 cognitions related to different themes (e.g., the self, the future, one’s own grief reactions). Respondents are instructed to rate the presence of each cognition (e.g., “Since s/he died, I think of myself as a weak person”) during the previous month, on four-point scales, with anchors 0 = *never* through 3 = *very often*. Factor analysis indicated that items form a single dimension of negative cognitions [10]. In the present sample, the Cronbach’s alpha was .92.

#### Children’s avoidance reactions

Avoidance was assessed using the Grief Behaviour Questionnaire for Children (GBQ-C). This is an adjusted version of the adult Depressive and Anxious Avoidance in Prolonged Grief Questionnaire [DAAPGQ; 30]. It measures both anxious avoidance (i.e., phobic avoidance of loss-related stimuli; e.g., “I avoid situations and places reminding me that s/he is dead”) and depressive avoidance (i.e., passivity and withdrawal from usual activities considered meaningless or unfulfilling; e.g., “I prefer to stay at home these days, rather than doing fun things with others”). Respondents rate the degree to which they engaged in each behaviour during the previous month on four-point scales (0 = *never*, 1 = *sometimes*, 2 = *often*, 3 = *very often*). Items measuring anxious and depressive avoidance yielded alphas of .81 and .85, respectively, and were summed to obtain indices of both forms of avoidance.

#### Children-rated parenting behaviours

Children completed two subscales of the Parenting Practices Questionnaire (PPQ), the first measuring warmth and involvement (11 items, e.g., “My mother/father shows sympathy when I am frustrated”) and the second assessing reasoning/induction (six items, e.g., “My mother/father explains the consequences of my behaviour”). The measure was developed by Robinson et al. [31] and has good psychometric properties [32]. Children rated to what extent items applied, on five-point scales, ranging from 1 (never) to 5 (always). Children also completed a shortened, seven item version of the 13-item autonomy-granting scale from the Mother-Father-Peer Scale [MFP; 33], to measure psychological autonomy granting, an aspect of positive parenting. Because of time constraints, a slightly shorter version of the scale was used. Children rated items (e.g., “My mother/father helps me to make my own decisions”) on four-point scales with anchors 1 = *not at all true* to 4 = *very true*. The inventory has adequate psychometric properties [34]. Children completed the scale twice, about both their parents/caregivers, when they were both alive, or only once when only one of them was present or alive. In the former case, scores of one of the parents/caregivers were randomly selected for inclusion in our analyses. (Specifically, for 34 children of all N = 159 included in this study, data were available from both caregivers. That is, in these cases, children reported about the parenting strategies of both parents and in these cases, both caregivers completed the CBCL and both parents reported about their own mental health and caregiver strategies. In these cases, one caregiver was randomly chosen whose data were used. This was the same caregiver for all the variables that we analysed.) Considering the data included in our analyses, internal consistencies for the PPQ warmth and involvement, PPQ reasoning/induction, and MFP autonomy granting scales were .90, .88, and .82 respectively.

#### Caregiver-rated internalizing and externalizing

Children’s levels of internalizing and externalizing problems as rated by their caregivers were assessed using the Child Behavior Checklist/6–18 [CBCL; 35]. The CBCL includes 118 items, measuring eight different problem areas (e.g., anxiety, depression, aggressive behaviour, attentional problems) that can be combined to obtain indices of (e.g.) Internalizing Problems and Externalizing Problems. Items are rated on three-point scales (0 = *not true*, 1 = *somewhat or sometimes true*, 2 = *very true/often true*). Psychometric properties of the original version [35] and Dutch version [36] are adequate. The internal consistencies of the Internalizing and Externalizing scales, considered in the current study, were .88 and .89 respectively.

#### Caregiver’s PGD symptoms

PGD symptoms of caregivers were assessed using the 30-item ICG-R [22]. It includes PGD symptoms as included in DSM-5-TR and ICD-11 and accompanying symptoms of unhealthy grief (e.g., “I feel I have trouble accepting the death.”). Participants rate the presence of symptoms during the previous month on five-point scales ranging from 1 = *never* to 5 = *always*. Psychometric properties of the Dutch ICG-R are good [37]. In the current sample, the α of the ICG-R selected for the analyses was .96.

#### Caregiver’s anxiety and depression

Caregivers completed the Hospital Anxiety and Depression Scale [HADS; 38] to rate the presence of seven depression symptoms (e.g., “I feel as if I am slowed down”) and seven anxiety symptoms (e.g., “I get sudden feelings of panic”) during the preceding week, on 4-point scales with different anchors. English [38] and Dutch versions [39] have adequate psychometric properties. In the current sample, the α’s of the HADS-depression and HADS-anxiety scales included in the analyses were .88 and .86, respectively.

#### Caregiver-rated parenting behaviours

Like the children, caregivers completed two subscales of the PPQ [31, 32], measuring warmth and involvement (11 items, e.g., “I show sympathy when my child is frustrated”) and reasoning/induction (six items, e.g., “I explain the consequences of his/her behaviour”), respectively. Items were rated on five-point scales, with anchors 1 = *never* and 5 = *always*. Like their children, caregivers also completed the seven item version of the autonomy-granting scale from the MFP [33, 34], to measure their perspective on psychological autonomy granting. Items (e.g., “I encourage him/her to make his/her own decisions”) were rated on four-point scales, with anchors 1 = *not at all true* to 4 = *very true*. In the current sample, the α’s of the scales PPQ warmth and involvement, PPQ reasoning/induction, and MFP autonomy granting as included in our analyses were .92, .90, and .86 respectively.

### Statistical analyses

The analyses we planned to address the study aims were conducted in six steps. In a first step, we examined if scores on the indices of children’s bereavement outcomes (prolonged grief (PG), depression, PTS, functional impairment, internalizing, and externalizing) differed as a function of the sociodemographic and loss-related characteristics of the sample (sex [male or female], age [in years], time since loss [in months], kinship to the deceased [lost person is parent, sibling, other close person], cause of death [illness, unnatural death, sudden medical death, unknown], and whether the death was expected/anticipated [yes or no]), using analysis of variance and Pearson correlations. These analyses were performed because we wished to examine the associations between children’s bereavement outcomes and the other variables considered, controlling for relevant sociodemographic and loss-related characteristics (i.e., the ones associated with one or more outcomes). In the second step, we examined associations between indices of children’s bereavement outcomes and the three cognitive behavioural variables using zero-order correlations. Additionally, we planned to use regression analyses to examine if cognitive behavioural variables that were significantly correlated with one or more of these outcomes, continued to be associated with these outcomes when controlling for the shared variance between these cognitive behavioural variables plus relevant sociodemographic and loss-related characteristics. In the third step, a similar procedure was followed with child-rated indices of parenting (i.e., warmth and involvement, reasoning/induction, autonomy granting) considered as correlates of children’s bereavement outcomes. In the fourth step, this procedure was repeated with caregiver’s mental health (i.e., their levels of PG, depression, and anxiety) considered as correlates of children’s bereavement outcomes. In the fifth step, this procedure was repeated with caregiver-rated indices of parenting considered as correlates of indices of children’s bereavement-related distress. In the sixth and last step of the analyses, we planned to perform a series of six regression analyses in which all six indices of children’s bereavement outcomes were consecutively regressed on all variables emerging as significant correlates in the previous steps, entered to the regression analyses simultaneously. There were occasional missing values which were handled using listwise deletion. When we ran analyses using multiple imputation, the same outcomes were found. Because we performed secondary analyses of data originally gathered for our RCT, we did not perform a-priori power analysis for this study. However, the sample size was sufficiently large to perform the analyses we planned, considering a power of .80, an alpha of .05, and the number of independent variables included in the regression analyses [cf. 40].

## Results

### Descriptive statistics and preliminary analyses

**Table 1** shows sociodemographic and loss-related characteristics of the sample. The scores on the measures of children’s bereavement outcomes (i.e., self-rated PG, depression, PTS, and PTS-related functional impairment, and caregiver-rated internalizing and externalizing) did not differ significantly as a function of any of these characteristics. For instance, correlations of age and time since loss with outcomes were all < .17; t-values for differences as a function of dichotomized sex and expectedness of death were all < 1.20; F-test for the ANOVAs examining differences as a function of kinship and cause were all < 2.45, and p-values for all statistical tests were > .05. Therefore, sociodemographic and loss-related characteristics were not considered in our further analyses.

### Associations of children’s bereavement outcomes with cognitive behavioural variables

**Table 2** shows zero-order correlations of the six children’s bereavement outcomes with indices of negative cognitions, anxious avoidance, and depressive avoidance. All correlations were statistically significant, except correlations with externalizing. Outcomes of the multiple regression analyses, with all children’s outcomes subsequently regressed on the three cognitive behavioural variables (entered simultaneously) are summarized in **S1 Table**. All five regression models were statistically significant. In the regression analyses with PG, depression, and PTS-related functional impairment as dependent variables, negative cognitions and depressive avoidance explained unique variance. In the regression predicting PTS total scores, all three cognitive behavioural variables explained unique variance. In the regression predicting internalizing, depressive avoidance explained unique variance. Notice that we did not control for any sociodemographic or loss-related variables in these regression analyses, nor in any of the other regression analyses we conducted, because none of the sociodemographic or loss-related variables were associated with the six children’s bereavement outcomes that we considered.

**Table 2:** Correlations between children’s bereavement outcomes and cognitive behavioural variables, caregiver’s mental health, and parenting behaviours.

	Prolonged grief (children-rated)	Depression (children-rated)	Posttraumatic stress (children-rated)	Functional impairment (children-rated)	Internalizing (caregiver-rated)	Externalizing (caregiver-rated)
**Cognitive behavioural variables**						
Negative cognitions	.76***	.63***	.75***	.48***	.16*	.07
Anxious avoidance	.62***	.55***	.66***	.34***	.21*	.04
Depressive avoidance	.64***	.66***	.68***	.48***	.26**	.05
**Caregiver’s mental health**						
Caregiver’s prolonged grief	.10	-.06	-.02	.07	.10	-.10
Caregiver’s depression	.08	-.02	.04	.21**	.12	-.08
Caregiver’s anxiety	.04	-.03	.03	.12	.25**	-.04
**Children-rated parenting behaviours**						
Children-rated warmth/involvement	.04	-.09	-.01	-.15	-.07	-.04
Children-rated reasoning/induction	.04	-.04	.03	-.11	.03	.15
Children-rated autonomy granting	.04	-.08	.05	-.04	-.07	< .01
**Caregiver-rated parenting behaviours**						
Caregiver-rated warmth/involvement	.06	-.07	< .01	-.08	-.04	.02
Caregiver-rated reasoning/induction	-.04	-.10	-.07	-.16*	< -.01	.15
Caregiver-rated autonomy granting	.03	< .01	.03	.04	.13	.07

Note.

* p < .05.

** p < .01.

*** p < .001.

### Associations of children’s bereavement outcomes with caregiver’s mental health

Zero-order correlations of children’s bereavement outcomes with caregiver’s mental health (**Table 2**) were all low and not statistically significant, except for a small correlation between PTS-related functional impairment and caregiver’s depression (r = .21, p = .009) and between Internalizing problems and caregiver’s anxiety (r = .25, p = .001). Not unexpectedly, when we regressed PTS functional impairment on caregiver’s PG, depression, and anxiety, entered simultaneously, PTS functional impairment continued to be associated with caregiver’s depression. Similarly, when Internalizing was regressed on all three indices of caregiver’s mental health, it continued to be associated with caregiver’s anxiety. These regression analyses are summarized in **S2 Table**.

For exploratory reasons, we examined if associations of children’s outcomes with caregiver’s mental health were moderated by whether the mental health was reported by the mother or the father (i.e., the source of the caregiver’s mental health index). To this end, we ran a series of 18 regression analyses in which the six children’s outcomes were consecutively regressed on each index of caregiver’s mental health (PG, depression, anxiety) plus the source of the parental information (mother or father), and the interaction between the mental health index and source. For instance, in a first regression, children’s PG was regressed on parent’s PG, source (mother or father), and the interaction between parent’s PG and source. Results are summarized in **S3–S5 Tables**. These results indicated that the associations between the children’s outcomes and caregiver’s mental health did not differ as a function of whether the caregiver was the reporting mother or the father.

### Associations of children’s bereavement outcomes with warmth/involvement, reasoning/induction, and autonomy granting rated by children

Zero-order correlations of all six children’s outcomes with the indices of parenting, as rated by the children were all low and statistically non-significant (**Table 2**). Again, for exploratory reasons, we ran a series of moderated regressions to examine if the associations of the six children’s outcomes with the three indices of parenting were qualified by whether children reported about their father or mother. Interestingly, several significant moderation effects were found (see **S6–S8 Tables**). The association of child-rated warmth and involvement (PPQ) with children’s PG was different for both sources (**S6 Table**). For children reporting about the mother’s warmth and involvement, the association with PG was positive and significant (r = .24, p = .018). For children reporting about the father’s warmth and involvement, the association with PG was negative and significant (r = -.29, p = .024). The associations of warmth and involvement with children’s depression and PTS were similarly moderated. For children reporting about their mother, warmth and involvement was unrelated to depression (r = .05, p = .611) and PTS (r = .15, p = .133). For children reporting about their father, more warmth and involvement was related with lower depression (r = -.31, p = .016) and PTS (r = -.29, p = .027). Associations of reasoning/induction (PPQ) with children’s distress were not moderated (**S7 Table**).

Some significant moderation effects were also found in the associations of children’s bereavement outcomes with child-rated autonomy granting (MFP; **S8 Table**). Association with children’s PG and PTS were moderated, yet the separate associations for mothers and fathers did not reach statistical significance. The association with children’s depression was moderated: For children reporting about the mother’s autonomy granting, the association with depression was not significant (r = .06, p = .577). For children reporting about the father’s autonomy granting, the association with PG was negative and significant (r = -.31, p = .017).

### Associations of children’s bereavement outcomes with warmth/involvement, reasoning/induction, and autonomy granting rated by caregivers

Zero-order correlations of children’s outcomes with indices of parenting rated by the caregivers were all low and statistically non-significant (**Table 2**), with one exception: more PTS- related functional impairment was associated with lower caregiver-rated reasoning/induction (r = -.16, p = .044). Not unexpectedly, when we regressed PTS functional impairment on caregiver-rated warmth/involvement, reasoning/induction, and autonomy granting entered simultaneously, lower caregiver-rated reasoning/induction continued to explain variance in PTS functional impairment, above and beyond parent-related warmth and involvement and autonomy (Beta = -0.22, t = -2.12, p = .035). For details, see **S9 Table**. We examined if associations were qualified by whether the parenting variables were reported by the mother or the father, in distinct regression analyses. As shown in **S10–S12 Tables**, this was not the case.

### Association of children’s bereavement outcomes with combinations of variables

We had planned to examine the degree to which variables emerging as significant correlates of the children’s outcomes in the univariate analyses (i.e., significant correlations reported in **Table 2**) explained unique variance in these indices when controlling for the shared variance between these indices, in multiple regressions. Children’s PG, depression, and PTS were only significantly correlated with the three cognitive behavioural variables (but not with the other variables). As summarized in **S1 Table** and reported above, negative cognitions and depressive avoidance emerged as unique correlates of PG and depression, and all three variables as unique correlates of PTS.

Children’s functional impairment associated with PTS was correlated with all three cognitive behavioural variables, caregiver’s depression, and caregiver-rated reasoning/induction. When we regressed functional impairment on all these variables, a significant model emerged (see **S13 Table**). Increased negative cognitions, depressive avoidance, and caregiver’s depression (but not anxious avoidance and reasoning/induction) explained unique variance in functional impairment. Children’s internalizing problems were correlated with all three cognitive behavioural variables and elevated caregiver’s anxiety. When we regressed internalizing on all these variables, a significant model emerged (see **S13 Table**). Increased depressive avoidance and caregiver’s anxiety (but not negative cognitions and anxious avoidance) contributed to elevated internalizing. We did not further consider externalizing, as none of the variables were associated with externalizing (**Table 2**).

## Discussion

When children are confronted with the death of a mother, father, sibling, or other close person, they face the challenge of adjusting to the loss in a situation where people close are also grieving. In this context, children are mourning their own loss and are also exposed to the pain of their family members. Conceivably, the children’s grief reactions are affected both by their individual way of coping and processing as well as by the emotional responses of caregivers and other systemic processes [cf. 9, 11–15, 41]. The current study sought to enhance knowledge about the relative importance of psychological (individual-level) and systemic (family-level) variables in affecting the severity of PGD symptoms and other outcomes in children confronted with the death of a loved one. In so doing, we focused specifically on negative cognitions and maladaptive avoidance behaviours [key mechanisms underlying PG from the perspective of cognitive behavioural grief theories; 10, 19] and emotional functioning and parenting behaviours of caregivers [central to system-based perspectives on grief, including Sandler’s et al.’s family bereavement program; 12, 13].

We first considered association of children’s bereavement outcomes with negative cognitions, anxious avoidance of loss-related cues, and depressive avoidance of potentially adaptive activities. All children’s self-reported bereavement outcomes (PG, depression, PTS, and functional impairment associated with PTS) and caregiver-rated internalizing were significantly correlated with these three cognitive behavioural variables. When we controlled for the shared variance between these variables, negative cognitions and depressive avoidance turned out to have the strongest relationship with most of these outcomes. That negative bereavement-related cognitions were associated with outcomes accords with prior studies [10]. That both forms of avoidance and particularly depressive avoidance were associated with bereavement outcomes is consistent with studies in adults [42, 43] and supports key principles from cognitive behavioural grief theories [19]. Interestingly, the cognitive behavioural variables were associated with caregiver-rated internalizing but not externalizing problems. Thus, to the extent that children display aggressive or oppositional behaviour following loss, this seems unrelated to their propensity to engage in negative thinking and avoidant coping.

We also examined associations of children’s PG and other bereavement outcomes with the severity of caregiver’s PG, depression, and anxiety. Strikingly, all associations were close to zero. Apparently, if caregivers experience more grief, depression, and anxiety, this does not mean that their children experience more emotional problems (and vice versa). These results are broadly in line with different studies in psychotraumatology showing that the intensity of parental distress and children’s distress in the face of traumatic life events are not, or only to a small degree, associated [16], especially when (as in our study) the distress was measured separately in parents and children and not reported exclusively by the parent. The absence of a linkage between children’s PG, depression, and PTS and caregiver’s PG, depression and anxiety is interesting. It may be interpreted as indicating that these emotional experiences in the face of loss have relatively independent, separate courses and determinants. Apparently, no transfer has occurred, where caregiver’s reactions spread to the children or vice versa [but see 41]. At the same time, it is possible that the association between children and caregiver responses is moderated by family characteristics (e.g., greater enmeshment) and other non-assessed variables or that transfer occurs for some, but not other outcomes. Indeed, we did observe two significant but small associations between children’s functional impairment and caregiver depression and between children’s internalizing and caregiver anxiety. The interaction between children and caregiver emotions in the face of loss is not well understood and warrants further scrutiny.

Another study aim was to examine to what extent children’s levels of PG and other bereavement outcomes were associated with parenting behaviours, as rated by children and by caregivers. Here, several notable findings emerged. First, considering the caregiver’s own ratings of their degree of warmth and involvement, reasoning and induction, and autonomy granting, we found that these ratings were all unrelated with children’s bereavement outcomes. There was one exception: there was a small but significant association between higher PTS-related functional impairment and lower caregiver-rated reasoning/induction. Considering the many correlations we considered, this finding should not be overrated. Yet it does provide a tentative indication that when children struggle with everyday activities (school, friends, home life) after loss, this may relate to caregivers being slightly less inclined to explain the consequences of the child’s behaviours to children. What is more striking is that the other indicators of caregiver-rated parenting behaviours were unrelated to children’s PG and the other outcomes. This finding is inconsistent with some system-based perspectives on grief, including Sandler’s et al.’s family bereavement program [12, 13] and is somewhat unexpected considering prior research among bereaved children indicating that higher positive parenting—which has some overlap with the parenting indices examined in this study—was indeed associated with lower mental health problems in parentally bereaved children [e.g., 44].

There are several possible explanations for differences between our and prior research. First, prior studies exclusively focused on parentally bereaved children, whereas our study included a more heterogenous group of children. Possibly, associations of parenting and children’s distress are moderated by who died within the family. Second, prior studies examined caregiver-rated internalizing and externalizing [e.g., 44] but not PG, depression, and PTS. It is possible that parenting has a different influence on different children’s outcomes. Third, it is possible that relatively well-functioning family systems were overrepresented in our sample; associations between parenting and children’s bereavement outcomes may be stronger in less healthy functioning family systems [cf. 9, 45]. Fourth, parenting behaviours are possibly more validly assessed using interview-based assessment, rather than questionnaire measures employed in this study. All this notwithstanding, the absence of a linkage of parenting and bereavement outcomes is not entirely unexpected considering research in PTS, showing that parenting generally shows a very weak association with children’s PTS following all kinds over adverse events [17]. Taken together, it seems imperative to further examine the role of parenting behaviours in children’s responses to bereavement, as well as possible moderators of this associations.

We also considered associations of children’s outcomes with their own observations of their caregiver’s warmth and involvement, reasoning and induction, and autonomy granting. Notably, when we considered correlations for the entire group, with some children reporting about their mother and others about their father, none of the children’s bereavement outcomes were associated with the parenting behaviours. For exploratory reasons, we examined the moderation effect of the caregiver the children reported about, considering that mothers and fathers may have different parenting behaviours with different impacts on their offspring. Associations of outcomes with reasoning and induction were not moderated. For warmth and involvement and for autonomy granting, interesting findings emerged. With respect to children-rated warmth/involvement, for children reporting about their mother, more warmth/involvement was associated with *more* PG. For children reporting about their father, more warmth was associated with *less* PG. Associations with depression and PTS also differed, with more warmth/involvement being associated with less depression and PTS among children reporting about their father, but not those reporting about their mother. With respect to children-rated autonomy granting, a similar significant moderation was found, with more autonomy granting being associated with less depression among children reporting about their father, but not those reporting about their mother. Again, in light of the many correlations we examined, we should be cautious not to overinterpret these outcomes. Broadly, the findings do, however, suggest that when—in the eyes of children—fathers display more warmth/involvement and grant more autonomy, this coincides with children experiencing lower grief complaints, whereas for mothers no such linkages exist. We can only speculate about the direction of causality in this association. It is possible that father’s display of positive parenting mitigated their children’s suffering. Alternatively, it is possible that when children experience less severe suffering, this encourages fathers to show more warmth and involvement and to grant more autonomy. It is also possible that the differential impact of father’s and mother’s parenting behaviours on children is affected by who died. In many cases that children reported about their mother’s parenting, the father had died, whereas in many cases children reported about their father’s parenting, the mother had passed away. Speculatively, it is possible that the connection between the remaining parent and the child for emotional support and parental advice changes much more strongly when mothers die than when fathers pass away.

In a final series of regression analyses, we had planned to examine which of the individual-level and family-level variables were most important correlates of the children’s bereavement outcomes. Because, however, apart from the cognitive behavioural variables, the family-level variables were not or hardly correlated with these outcomes, this added little to the rest of our findings. Children’s PG, depression, and PTS were correlated with the individual-level cognitive behavioural variables, but not with the family-level variables. Greater functional impairment was associated with negative cognitions, depressive avoidance, and caregiver’s depression. Internalizing was associated with increased depressive avoidance and caregiver’s anxiety. Externalizing was not associated with any of the individual-levels and family-levels variables considered.

There are several limitations that should be considered when interpreting the present outcomes. First and foremost, this was a cross-sectional study and, consequently, no conclusions can be drawn about the direction of causality between variables. So, for instance, future research is needed to examine whether negative cognitions and avoidant coping are, like in adults [46], associated with outcomes assessed later in time. Moreover, as noted above, the temporal order of the association between father’s parenting behaviours and children’s outcomes that we observed warrants further scrutiny. Second, only few family-level variables were assessed. If would be interesting for future studies to examine to what extent other variables (e.g., family communication, cohesion) moderate and mediate the association between children’s and caregiver’s emotional responses to bereavement [cf. 45]. Third, data were all collected using questionnaire measures. Research in PTS indicated that associations between children’s and parent’s PTS are stronger when interview-based assessment is used [16]. Accordingly, associations we found between mental health and parenting as reported by children and caregivers may have been underestimated because of our use of questionnaires. Future research should preferably use interview-based assessments.

## Conclusion

Notwithstanding these limitations, the present study adds to a gradually growing literature examining associations between children’s responses to loss and responses of their caregivers. Findings suggest that associations of children’s symptom-levels of PG and associated indices of functioning with responses of caregivers may be small, and that children’s responses are more strongly driven by individual cognitive behavioural processes. In terms of clinical implications, these findings indicate that, when helping children who are stuck in processing loss, it is important to pay close attention to the extent to which they have negative, rigid, unhelpful thinking patterns and to address these with cognitive restructuring (including challenging cognitions and behavioural experiments). Likewise, it is indicated to help them reduce anxious avoidance of loss-related stimuli and to redirect depressive avoidance into active behaviours that help incorporate the loss into life. However, also considering prior research and theorizing pointing at the relevance of family processes in children’s responses to loss, more research on the interaction between children’s grief and the emotional responses and parenting behaviours of their caregivers and on the interplay between individual cognitive behavioural processes and systemic influences is urgently needed. If future research shows that children’s reactions are indeed significantly affected by the responses of their caregivers as well as other family-level variables, that may have clinical implications. For instance, it would emphasize the importance of considering the influence of caregiver’s reactions, parenting styles, and family-characteristics (e.g., cohesion, communication) in the assessment of children’s grief reactions, in order to capture a broad range of factors affecting the children’s responses. In addition, it would suggest the importance of addressing systemic and family-level variables in the treatment of children’s PGD. For instance, in instances were caregiver’s grief-related distress is considered to interfere with the children’s grief, parental counselling could be offered parallel to individual treatment of children. Observations in our own clinical trial indicated that caregiver counselling was a welcome addition to the individual CBT for children. In cases where the children’s grief is hindered by problematic family functioning (e.g., poor communication, conflict regulation, cohesion) family-focused interventions may be used. More research is urgently needed to enhance knowledge about the interaction between grief reactions of children and their caregivers, to inform the development of interventions promoting adaptive grieving in children.

## Supporting information

S1 Table Regression analyses with indices of children’s bereavement-related distress regressed on cognitive behavioural variables. (DOCX)

S2 Table Regression analyses with PTS functional impairment and internalizing regressed on indices of caregiver’s mental health. (DOCX)

S3 Table Regression analyses with children’s bereavement outcomes regressed on caregiver’s prolonged grief, source of caregiver’s information, and their interaction. (DOCX)

S4 Table Regression analyses with children’s bereavement outcomes regressed on caregiver’s depression, source of caregiver’s information, and their interaction. (DOCX)

S5 Table Regression analyses with children’s bereavement outcomes regressed on caregiver’s anxiety, source of caregiver’s information, and their interaction. (DOCX)

S6 Table Regression analyses with children’s bereavement outcomes regressed on children-rated warmth/involvement, source of caregiver’s information, and their interaction. (DOCX)

S7 Table Regression analyses with children’s bereavement outcomes regressed on children-rated reasoning/induction, source of caregiver’s information, and their interaction. (DOCX)

S8 Table Regression analyses with children’s bereavement outcomes regressed on children-rated autonomy granting, source of caregiver’s information, and their interaction. (DOCX)

S9 Table Regression analysis with PTS functional impairment regressed on caregiver-rated indices of parenting. (DOCX)

S10 Table Regression analyses with children’s bereavement outcomes regressed on caregiver-rated warmth/involvement, source of caregiver’s information, and their interaction. (DOCX)

S11 Table Regression analyses with children’s bereavement outcomes regressed on caregiver-rated reasoning/induction, source of caregiver’s information, and their interaction. (DOCX)

S12 Table Regression analyses with children’s bereavement outcomes regressed on caregiver-rated autonomy granting, source of caregiver’s information, and their interaction. (DOCX)

S13 Table Regression analyses with individual and systemic variables predicting PTS functional impairment and internalizing. (DOCX)
