# Supplementary material for: Individual and systemic variables associated with prolonged grief and other emotional distress in bereaved children
Source: PLoS One. 2024 Apr 30;19(4):e0302725. doi: 10.1371/journal.pone.0302725 (PMC11060573; doi:10.1371/journal.pone.0302725)
Supplement: S1 Table — (DOCX) [file pone.0302725.s001.docx]

**Supporting Information Table 1**

Regression analyses with indices of children’s bereavement-related distress regressed on cognitive behavioural variables

|  | B | SE B | β | F | DF | *R*^2^ |
| --- | --- | --- | --- | --- | --- | --- |
| DV = Children’s prolonged grief |  |  |  | 77.95*** | 3, 157 | .60 |
| Negative cognitions | 0.609 | 0.093 | .559*** |  |  |  |
| Anxious avoidance | 0.295 | 0.178 | .123 |  |  |  |
| Depressive avoidance | 0.532 | 0.256 | .158* |  |  |  |
| DV = Children’s depression |  |  |  | 49.46*** | 3, 157 | .49 |
| Negative cognitions | 0.176 | 0.068 | .249* |  |  |  |
| Anxious avoidance | 0.185 | 0.131 | .119 |  |  |  |
| Depressive avoidance | 0.884 | 0.187 | .406*** |  |  |  |
| DV = Children’s posttraumatic stress |  |  |  | 82.14*** | 3, 157 | .62 |
| Negative cognitions | 0.387 | 0.076 | .425*** |  |  |  |
| Anxious avoidance | 0.404 | 0.146 | .202** |  |  |  |
| Depressive avoidance | 0.684 | 0.210 | .243*** |  |  |  |
| DV = Children’s functional impairment linked with posttraumatic stress |  |  |  | 19.09*** | 3, 157 | .27 |
| Negative cognitions | 0.055 | 0.020 | .320** |  |  |  |
| Anxious avoidance | -0.026 | 0.038 | -.070 |  |  |  |
| Depressive avoidance | 0.154 | 0.054 | .292** |  |  |  |
| DV = Caregiver-rated internalizing |  |  |  | 4.18** | 3, 156 | .08 |
| Negative cognitions | -0.106 | 0.107 | -.128 |  |  |  |
| Anxious avoidance | 0.233 | 0.207 | .128 |  |  |  |
| Depressive avoidance | 0.693 | 0.296 | .272* |  |  |  |

Note. DV = Dependent variable.

* p < .05. ** p < .01. *** p < .001
