# Supplementary material for: Individual and systemic variables associated with prolonged grief and other emotional distress in bereaved children
Source: PLoS One. 2024 Apr 30;19(4):e0302725. doi: 10.1371/journal.pone.0302725 (PMC11060573; doi:10.1371/journal.pone.0302725)
Supplement: S2 Table — (DOCX) [file pone.0302725.s002.docx]

**Supporting Information Table 2**

Regression analyses with PTS functional impairment and internalizing regressed on indices of caregiver’s mental health

|  | B | SE B | β | F | DF | *R*^2^ |
| --- | --- | --- | --- | --- | --- | --- |
| DV = Children’s functional impairment linked with posttraumatic stress |  |  |  | 3.44* | 3, 154 | .06 |
| Caregiver’s prolonged grief | -0.021 | 0.011 | -.251 |  |  |  |
| Caregiver’s depression | 0.149 | 0.053 | .366** |  |  |  |
| Caregiver’s anxiety | 0.021 | 0.052 | .048 |  |  |  |
| DV = Caregiver-rated internalizing |  |  |  | 4.24** | 3, 153 | .08 |
| Caregiver’s prolonged grief | -0.060 | 0.054 | -.150 |  |  |  |
| Caregiver’s depression | -0.074 | 0.253 | -.038 |  |  |  |
| Caregiver’s anxiety | 0.812 | 0.250 | .387** |  |  |  |

Note. DV = Dependent variable. PTS = Posttraumatic stress.

* p < .05. ** p < .01. *** p < .001.
