# Supplementary material for: Individual and systemic variables associated with prolonged grief and other emotional distress in bereaved children
Source: PLoS One. 2024 Apr 30;19(4):e0302725. doi: 10.1371/journal.pone.0302725 (PMC11060573; doi:10.1371/journal.pone.0302725)
Supplement: S3 Table — (DOCX) [file pone.0302725.s003.docx]

**Supporting Information Table 3**

Regression analyses with children’s bereavement outcomes regressed on caregiver’s prolonged grief, source of caregiver’s information, and their interaction

|  | B | SE B | β | F | DF | *R*^2^ |
| --- | --- | --- | --- | --- | --- | --- |
| DV = Children’s prolonged grief |  |  |  | 1.13 | 3, 154 | .022 |
| Caregiver’s prolonged grief | 0.079 | 0.056 | .151 |  |  |  |
| Source | 2.201 | 5.569 | .093 |  |  |  |
| Interaction Caregiver’s PG x Source | -0.069 | 0.086 | -.191 |  |  |  |
| DV = Children’s depression |  |  |  | 0.57 | 3, 154 | .011 |
| Caregiver’s prolonged grief | -0.004 | 0.037 | -.012 |  |  |  |
| Source | 1.542 | 3.685 | .099 |  |  |  |
| Interaction Caregiver’s PG x Source | -0.042 | 0.057 | -.178 |  |  |  |
| DV = Children’s posttraumatic stress |  |  |  | 0.44 | 3, 154 | .009 |
| Caregiver’s prolonged grief | -0.003 | 0.047 | -.008 |  |  |  |
| Source | -0.680 | 4.657 | -.035 |  |  |  |
| Interaction Caregiver’s PG x Source | -0.018 | 0.072 | -.060 |  |  |  |
| DV = Children’s functional impairment linked with posttraumatic stress |  |  |  | 0.33 | 3, 154 | .007 |
| Caregiver’s prolonged grief | 0.008 | 0.009 | .095 |  |  |  |
| Source | 0.434 | 0.878 | .117 |  |  |  |
| Interaction Caregiver’s PG x Source | -0.005 | 0.014 | -.085 |  |  |  |
| DV = Caregiver-rated internalizing |  |  |  | 0.65 | 3, 154 | .013 |
| Caregiver’s prolonged grief | 0.032 | 0.043 | .082 |  |  |  |
| Source | -0.697 | 4.244 | -.039 |  |  |  |
| Interaction Caregiver’s PG x Source | 0.023 | 0.066 | .085 |  |  |  |
| DV = Caregiver-rated externalizing |  |  |  | 0.53 | 3, 153 | .015 |
| Caregiver’s prolonged grief | -0.023 | 0.042 | -.059 |  |  |  |
| Source | 1.313 | 4.133 | .075 |  |  |  |
| Interaction Caregiver’s PG x Source | -0.038 | 0.064 | -.142 |  |  |  |

Note. DV = Dependent variable. PG = Prolonged grief.

* p < .05. ** p < .01. *** p < .001.
