# Supplementary material for: Individual and systemic variables associated with prolonged grief and other emotional distress in bereaved children
Source: PLoS One. 2024 Apr 30;19(4):e0302725. doi: 10.1371/journal.pone.0302725 (PMC11060573; doi:10.1371/journal.pone.0302725)
Supplement: S4 Table — (DOCX) [file pone.0302725.s004.docx]

**Supporting Information Table 4**

Regression analyses with children’s bereavement outcomes regressed on caregiver’s depression, source of caregiver’s information, and their interaction

|  | B | SE B | β | F | DF | *R*^2^ |
| --- | --- | --- | --- | --- | --- | --- |
| DV = Children’s prolonged grief |  |  |  | 0.65 | 3, 155 | .013 |
| Caregiver’s depression | 0.223 | 0.256 | .085 |  |  |  |
| Source | -1.388 | 2.839 | -.058 |  |  |  |
| Interaction Caregiver’s depression x Source | -0.115 | 0.458 | -.032 |  |  |  |
| DV = Children’s depression |  |  |  | 0.32 | 3, 155 | .006 |
| Caregiver’s depression | 0.010 | 0.167 | .006 |  |  |  |
| Source | -0.149 | 1.854 | -.010 |  |  |  |
| Interaction Caregiver’s depression x Source | -0.176 | 0.299 | -.075 |  |  |  |
| DV = Children’s posttraumatic stress |  |  |  | 0.48 | 3, 155 | .009 |
| Caregiver’s depression | 0.145 | 0.213 | .067 |  |  |  |
| Source | -0.575 | 2.355 | -.029 |  |  |  |
| Interaction Caregiver’s depression x Source | -0.204 | 0.380 | -.069 |  |  |  |
| DV = Children’s functional impairment linked with posttraumatic stress |  |  |  | 2.97* | 3, 155 | .055 |
| Caregiver’s depression | 0.114 | 0.039 | .278** |  |  |  |
| Source | 0.591 | 0.432 | .159 |  |  |  |
| Interaction Caregiver’s depression x Source | -0.088 | 0.070 | -.157 |  |  |  |
| DV = Caregiver-rated internalizing |  |  |  | 1.02 | 3, 154 | .020 |
| Caregiver’s depression | 0.137 | 0.192 | .070 |  |  |  |
| Source | -0.676 | 2.125 | -.038 |  |  |  |
| Interaction Caregiver’s depression x Source | 0.302 | 0.342 | .113 |  |  |  |
| DV = Caregiver-rated externalizing |  |  |  | 0.539 | 3, 154 | .011 |
| Caregiver’s depression | -0.128 | 0.193 | -.065 |  |  |  |
| Source | -0.672 | 2.134 | -.038 |  |  |  |
| Interaction Caregiver’s depression x Source | -0.104 | 0.344 | -.039 |  |  |  |

Note. DV = Dependent variable.

* p < .05. ** p < .01. *** p < .001.
