# Supplementary material for: Individual and systemic variables associated with prolonged grief and other emotional distress in bereaved children
Source: PLoS One. 2024 Apr 30;19(4):e0302725. doi: 10.1371/journal.pone.0302725 (PMC11060573; doi:10.1371/journal.pone.0302725)
Supplement: S5 Table — (DOCX) [file pone.0302725.s005.docx]

**Supporting Information Table 5**

Regression analyses with children’s bereavement outcomes regressed on caregiver’s anxiety, source of caregiver’s information, and their interaction

|  | B | SE B | β | F | DF | *R*^2^ |
| --- | --- | --- | --- | --- | --- | --- |
| DV = Children’s prolonged grief |  |  |  | 0.45 | 3, 155 | .009 |
| Caregiver’s anxiety | 0.143 | 0.298 | .051 |  |  |  |
| Source | -0.869 | 3.560 | -.036 |  |  |  |
| Interaction Caregiver’s anxiety x Source | -0.169 | 0.462 | -.058 |  |  |  |
| DV = Children’s depression |  |  |  | 0.26 | 3, 155 | .005 |
| Caregiver’s anxiety | -0.056 | 0.194 | -.031 |  |  |  |
| Source | -0.744 | 2.322 | -.048 |  |  |  |
| Interaction Caregiver’s anxiety x Source | -0.039 | 0.301 | -.020 |  |  |  |
| DV = Children’s posttraumatic stress |  |  |  | 0.41 | 3, 155 | .008 |
| Caregiver’s anxiety | 0.127 | 0.247 | .055 |  |  |  |
| Source | -0.399 | 2.950 | -.020 |  |  |  |
| Interaction Caregiver’s anxiety x Source | -0.170 | 0.383 | -.070 |  |  |  |
| DV = Children’s functional impairment linked with posttraumatic stress |  |  |  | 1.04 | 3, 155 | .020 |
| Caregiver’s anxiety | 0.076 | 0.046 | .173 |  |  |  |
| Source | 0.506 | 0.550 | .136 |  |  |  |
| Interaction Caregiver’s anxiety x Source | -0.049 | 0.071 | -.106 |  |  |  |
| DV = Caregiver-rated internalizing |  |  |  | 3.69 | 3, 154 | .068* |
| Caregiver’s anxiety | 0.522 | 0.218 | .249* |  |  |  |
| Source | 0.719 | 2.601 | .040 |  |  |  |
| Interaction Caregiver’s anxiety x Source | 0.063 | 0.337 | .029 |  |  |  |
| DV = Caregiver-rated externalizing |  |  |  | 0.32 | 3, 154 | .006 |
| Caregiver’s anxiety | -0.147 | 0.225 | -.070 |  |  |  |
| Source | -1.756 | 2.684 | -.098 |  |  |  |
| Interaction Caregiver’s anxiety x Source | 0.094 | 0.347 | .043 |  |  |  |

Note. DV = Dependent variable.

* p < .05. ** p < .01. *** p < .001.
