# Supplementary material for: Individual and systemic variables associated with prolonged grief and other emotional distress in bereaved children
Source: PLoS One. 2024 Apr 30;19(4):e0302725. doi: 10.1371/journal.pone.0302725 (PMC11060573; doi:10.1371/journal.pone.0302725)
Supplement: S6 Table — (DOCX) [file pone.0302725.s006.docx]

**Supporting Information Table 6**

Regression analyses with children’s bereavement outcomes regressed on children-rated warmth/involvement, source of caregiver’s information, and their interaction

|  | B | SE B | β | F | DF | *R*^2^ |
| --- | --- | --- | --- | --- | --- | --- |
| DV = Children’s prolonged grief |  |  |  | 4.08** | 3, 158 | .073 |
| Children-rated warmth/involvement | 0.313 | 0.130 | .250* |  |  |  |
| Source | 23.979 | 7.980 | 1.005** |  |  |  |
| Interaction | -0.660 | 0.198 | -1.099* |  |  |  |
| DV = Children’s depression |  |  |  | 2.52 | 3, 158 | .047 |
| Children-rated warmth/involvement | 0.042 | 0.085 | .052 |  |  |  |
| Source | 10.401 | 5.227 | .675* |  |  |  |
| Interaction | -0.297 | 0.130 | -.766* |  |  |  |
| DV = Children’s posttraumatic stress |  |  |  | 2.96* | 3, 158 | .054 |
| Children-rated warmth/involvement | 0.163 | 0.109 | .158 |  |  |  |
| Source | 15.771 | 6.667 | .799* |  |  |  |
| Interaction | -0.451 | 0.166 | -.909** |  |  |  |
| DV = Children’s functional impairment linked with posttraumatic stress |  |  |  | 1.42 | 3, 158 | .027 |
| Children-rated warmth/involvement | -0.020 | 0.021 | -.102 |  |  |  |
| Source | 0.903 | 1.268 | .244 |  |  |  |
| Interaction | -0.023 | 0.031 | -.244 |  |  |  |
| DV = Caregiver-rated internalizing |  |  |  | 0.49 | 3, 157 | .009 |
| Children-rated warmth/involvement | -0.009 | 0.103 | -.009 |  |  |  |
| Source | 5.288 | 6.299 | .291 |  |  |  |
| Interaction | -0.123 | 0.157 | -.269 |  |  |  |
| DV = Caregiver-rated externalizing |  |  |  | 0.34 | 3, 157 | .007 |
| Children-rated warmth/involvement | -0.016 | 0.100 | -.017 |  |  |  |
| Source | 1.857 | 6.134 | .105 |  |  |  |
| Interaction | -0.074 | 0.152 | -.166 |  |  |  |

Note. DV = Dependent variable.

* p < .05. ** p < .01. *** p < .001.
