# Supplementary material for: Individual and systemic variables associated with prolonged grief and other emotional distress in bereaved children
Source: PLoS One. 2024 Apr 30;19(4):e0302725. doi: 10.1371/journal.pone.0302725 (PMC11060573; doi:10.1371/journal.pone.0302725)
Supplement: S7 Table — (DOCX) [file pone.0302725.s007.docx]

**Supporting Information Table 7**

Regression analyses with children’s bereavement outcomes regressed on children-rated reasoning/induction, source of caregiver’s information, and their interaction

|  | B | SE B | β | F | DF | *R*^2^ |
| --- | --- | --- | --- | --- | --- | --- |
| DV = Children’s prolonged grief |  |  |  | 1.69 | 3, 158 | .032 |
| Children-rated reasoning/induction | 0.358 | 0.216 | .174 |  |  |  |
| Source | 11.488 | 7.110 | .482 |  |  |  |
| Interaction | -0.639 | 0.328 | -.590 |  |  |  |
| DV = Children’s depression |  |  |  | 0.41 | 3, 158 | .008 |
| Children-rated reasoning/induction | -0.011 | 0.141 | -.008 |  |  |  |
| Source | 1.496 | 4.648 | .097 |  |  |  |
| Interaction | -0.118 | 0.214 | -.169 |  |  |  |
| DV = Children’s posttraumatic stress |  |  |  | 1.02 | 3, 158 | .019 |
| Children-rated reasoning/induction | 0.198 | 0.179 | .117 |  |  |  |
| Source | 5.543 | 5.918 | .281 |  |  |  |
| Interaction | -0.349 | 0.273 | -.389 |  |  |  |
| DV = Children’s functional impairment linked with posttraumatic stress |  |  |  | 0.85 | 3, 158 | .016 |
| Children-rated reasoning/induction | -0.026 | 0.034 | -.082 |  |  |  |
| Source | 0.631 | 1.111 | .171 |  |  |  |
| Interaction | -0.026 | 0.051 | -.155 |  |  |  |
| DV = Caregiver-rated internalizing |  |  |  | 0.13 | 3, 157 | .002 |
| Children-rated reasoning/induction | 0.058 | 0.167 | .037 |  |  |  |
| Source | 1.071 | 5.509 | .059 |  |  |  |
| Interaction | -0.018 | 0.254 | -.021 |  |  |  |
| DV = Caregiver-rated externalizing |  |  |  | 1.33 | 3, 157 | .025 |
| Children-rated reasoning/induction | 0.222 | 0.161 | .146 |  |  |  |
| Source | -1.124 | 5.296 | -.064 |  |  |  |
| Interaction | 0.018 | 0.244 | .023 |  |  |  |

Note. DV = Dependent variable.
