# Supplementary material for: Individual and systemic variables associated with prolonged grief and other emotional distress in bereaved children
Source: PLoS One. 2024 Apr 30;19(4):e0302725. doi: 10.1371/journal.pone.0302725 (PMC11060573; doi:10.1371/journal.pone.0302725)
Supplement: S8 Table — (DOCX) [file pone.0302725.s008.docx]

**Supporting Information Table 8**

Regression analyses with children’s bereavement outcomes regressed on children-rated autonomy granting, source of caregiver’s information, and their interaction

|  | B | SE B | β | F | DF | *R*^2^ |
| --- | --- | --- | --- | --- | --- | --- |
| DV = Children’s prolonged grief |  |  |  | 2.37 | 3, 158 | .044 |
| Children-rated autonomy granting | 0.492 | 0.271 | .179 |  |  |  |
| Source | 21.291 | 9.731 | .893* |  |  |  |
| Interaction | -1.095 | 0.452 | -.984* |  |  |  |
| DV = Children’s depression |  |  |  | 2.49 | 3, 158 | .046 |
| Children-rated autonomy granting | 0.095 | 0.175 | .054 |  |  |  |
| Source | 13.557 | 6.277 | .880* |  |  |  |
| Interaction | -0.693 | 0.292 | -.964* |  |  |  |
| DV = Children’s posttraumatic stress |  |  |  | 2.63 | 3, 158 | .048 |
| Children-rated autonomy granting | 0.436 | 0.224 | .192 |  |  |  |
| Source | 17.972 | 8.028 | .911* |  |  |  |
| Interaction | -0.931 | 0.373 | -1.011* |  |  |  |
| DV = Children’s functional impairment linked with posttraumatic stress |  |  |  | 1.25 | 3, 158 | .024 |
| Children-rated autonomy granting | 0.031 | 0.042 | .072 |  |  |  |
| Source | 2.841 | 1.524 | .768 |  |  |  |
| Interaction | -0.130 | 0.071 | -.754 |  |  |  |
| DV = Caregiver-rated internalizing |  |  |  | 0.46 | 3, 157 | .009 |
| Children-rated autonomy granting | -0.047 | 0.211 | -.022 |  |  |  |
| Source | 5.830 | 7.563 | .321 |  |  |  |
| Interaction | -0.250 | 0.351 | -.295 |  |  |  |
| DV = Caregiver-rated externalizing |  |  |  | 0.13 | 3, 157 | .003 |
| Children-rated autonomy granting | -0.015 | 0.206 | -.007 |  |  |  |
| Source | -1.616 | 7.379 | -.091 |  |  |  |
| Interaction | 0.035 | 0.343 | .042 |  |  |  |

Note. DV = Dependent variable.

* p < .05. ** p < .01. *** p < .001.
