# Supplementary material for: Individual and systemic variables associated with prolonged grief and other emotional distress in bereaved children
Source: PLoS One. 2024 Apr 30;19(4):e0302725. doi: 10.1371/journal.pone.0302725 (PMC11060573; doi:10.1371/journal.pone.0302725)
Supplement: S9 Table — (DOCX) [file pone.0302725.s009.docx]

**Supporting Information Table 9**

Regression analysis with PTS functional impairment regressed on caregiver-rated indices of parenting

|  | B | SE B | β | F | DF | *R*^2^ |
| --- | --- | --- | --- | --- | --- | --- |
| DV = Children’s functional impairment linked with posttraumatic stress |  |  |  | 2.28* | 3, 156 | .043 |
| Caregiver-rated warmth and involvement | 0.008 | 0.028 | .036 |  |  |  |
| Caregiver-rated reasoning/induction | -0.096 | 0.045 | -.253* |  |  |  |
| Caregiver-rated autonomy granting | 0.060 | 0.040 | .139 |  |  |  |

Note. DV = Dependent variable. PTS = Posttraumatic stress.

* p < .10.
