# Supplementary material for: Individual and systemic variables associated with prolonged grief and other emotional distress in bereaved children
Source: PLoS One. 2024 Apr 30;19(4):e0302725. doi: 10.1371/journal.pone.0302725 (PMC11060573; doi:10.1371/journal.pone.0302725)
Supplement: S10 Table — (DOCX) [file pone.0302725.s010.docx]

**Supporting Information Table 10**

Regression analyses with children’s bereavement outcomes regressed on caregiver-rated warmth/involvement, source of caregiver’s information, and their interaction

|  | B | SE B | β | F | DF | *R*^2^ |
| --- | --- | --- | --- | --- | --- | --- |
| DV = Children’s prolonged grief |  |  |  | 0.69 | 3, 156 | .013 |
| Caregiver-rated warmth/involvement | 0.177 | 0.164 | .116 |  |  |  |
| Source | 9.235 | 11.427 | .387 |  |  |  |
| Interaction | -0.245 | 0.259 | -.440 |  |  |  |
| DV = Children’s depression |  |  |  | 0.54 | 3, 156 | .010 |
| Caregiver-rated warmth/involvement | -0.080 | 0.106 | -.081 |  |  |  |
| Source | -0.447 | 7.371 | -.029 |  |  |  |
| Interaction | -0.017 | 0.167 | -.048 |  |  |  |
| DV = Children’s posttraumatic stress |  |  |  | 0.63 | 3, 156 | .012 |
| Caregiver-rated warmth/involvement | 0.054 | 0.136 | .043 |  |  |  |
| Source | 6.433 | 9.444 | .326 |  |  |  |
| Interaction | -0.188 | 0.214 | -.409 |  |  |  |
| DV = Children’s functional impairment linked with posttraumatic stress |  |  |  | 0.36 | 3, 156 | .007 |
| Caregiver-rated warmth/involvement | -0.018 | 0.025 | -.076 |  |  |  |
| Source | 0.124 | 1.771 | .034 |  |  |  |
| Interaction | -0.002 | 0.040 | -.019 |  |  |  |
| DV = Caregiver-rated internalizing |  |  |  | 0.24 | 3, 155 | .005 |
| Caregiver-rated warmth/involvement | -0.079 | 0.127 | -.068 |  |  |  |
| Source | -4.620 | 8.793 | -.253 |  |  |  |
| Interaction | 0.121 | 0.200 | .284 |  |  |  |
| DV = Caregiver-rated externalizing |  |  |  | 0.37 | 3, 155 | .007 |
| Caregiver-rated warmth/involvement | -0.055 | 0.122 | -.048 |  |  |  |
| Source | -7.007 | 8.507 | -.396 |  |  |  |
| Interaction | 0.136 | 0.193 | .329 |  |  |  |

Note. DV = Dependent variable.
