# Supplementary material for: Individual and systemic variables associated with prolonged grief and other emotional distress in bereaved children
Source: PLoS One. 2024 Apr 30;19(4):e0302725. doi: 10.1371/journal.pone.0302725 (PMC11060573; doi:10.1371/journal.pone.0302725)
Supplement: S11 Table — (DOCX) [file pone.0302725.s011.docx]

**Supporting Information Table 11**

Regression analyses with children’s bereavement outcomes regressed on caregiver-rated reasoning/induction, source of caregiver’s information, and their interaction

|  | B | SE B | β | F | DF | *R*^2^ |
| --- | --- | --- | --- | --- | --- | --- |
| DV = Children’s prolonged grief |  |  |  | 0.41 | 3, 156 | .008 |
| Caregiver-rated reasoning/induction | -0.098 | 0.269 | -.040 |  |  |  |
| Source | -0.156 | 9.861 | -.007 |  |  |  |
| Interaction | -0.075 | 0.407 | -.075 |  |  |  |
| DV = Children’s depression |  |  |  | 0.86 | 3, 156 | .017 |
| Caregiver-rated reasoning/induction | -0.225 | 0.172 | -.142 |  |  |  |
| Source | -3.512 | 6.325 | -.229 |  |  |  |
| Interaction | 0.099 | 0.261 | .153 |  |  |  |
| DV = Children’s posttraumatic stress |  |  |  | 0.76 | 3, 156 | .015 |
| Caregiver-rated reasoning/induction | -0.166 | 0.221 | -.082 |  |  |  |
| Source | -1.156 | 8.118 | -.059 |  |  |  |
| Interaction | -0.034 | 0.335 | -.041 |  |  |  |
| DV = Children’s functional impairment linked with posttraumatic stress |  |  |  | 1.35 | 3, 156 | .026 |
| Caregiver-rated reasoning/induction | -0.061 | 0.041 | -.160 |  |  |  |
| Source | 0.011 | 1.510 | .003 |  |  |  |
| Interaction | 0.000 | 0.062 | .001 |  |  |  |
| DV = Caregiver-rated internalizing |  |  |  | 0.11 | 3, 155 | .002 |
| Caregiver-rated reasoning/induction | -0.032 | 0.207 | -.017 |  |  |  |
| Source | -1.276 | 7.578 | -.070 |  |  |  |
| Interaction | 0.086 | 0.313 | .112 |  |  |  |
| DV = Caregiver-rated externalizing |  |  |  | 1.34 | 3, 155 | .026 |
| Caregiver-rated reasoning/induction | 0.200 | 0.198 | .110 |  |  |  |
| Source | -4.086 | 7.254 | -.231 |  |  |  |
| Interaction | 0.145 | 0.299 | .195 |  |  |  |

Note. DV = Dependent variable.
