# Supplementary material for: Individual and systemic variables associated with prolonged grief and other emotional distress in bereaved children
Source: PLoS One. 2024 Apr 30;19(4):e0302725. doi: 10.1371/journal.pone.0302725 (PMC11060573; doi:10.1371/journal.pone.0302725)
Supplement: S12 Table — (DOCX) [file pone.0302725.s012.docx]

**Supporting Information Table 12**

Regression analyses with children’s bereavement outcomes regressed on caregiver-rated autonomy granting, source of caregiver’s information, and their interaction

|  | B | SE B | β | F | DF | *R*^2^ |
| --- | --- | --- | --- | --- | --- | --- |
| DV = Children’s prolonged grief |  |  |  | 0.31 | 3, 156 | .006 |
| Caregiver-rated autonomy granting | 0.068 | 0.266 | .024 |  |  |  |
| Source | -2.619 | 10.801 | -.110 |  |  |  |
| Interaction | 0.045 | 0.503 | .041 |  |  |  |
| DV = Children’s depression |  |  |  | 0.17 | 3, 156 | .003 |
| Caregiver-rated autonomy granting | -0.018 | 0.172 | -.010 |  |  |  |
| Source | -2.269 | 6.967 | -.148 |  |  |  |
| Interaction | 0.068 | 0.325 | .094 |  |  |  |
| DV = Children’s posttraumatic stress |  |  |  | 0.39 | 3, 156 | .008 |
| Caregiver-rated autonomy granting | 0.062 | 0.220 | .027 |  |  |  |
| Source | -1.833 | 8.914 | -.093 |  |  |  |
| Interaction | 0.011 | 0.415 | .012 |  |  |  |
| DV = Children’s functional impairment linked with posttraumatic stress |  |  |  | 0.18 | 3, 156 | .003 |
| Caregiver-rated autonomy granting | 0.009 | 0.041 | .021 |  |  |  |
| Source | -0.439 | 1.671 | -.119 |  |  |  |
| Interaction | 0.027 | 0.078 | .158 |  |  |  |
| DV = Caregiver-rated internalizing |  |  |  | 0.95 | 3, 155 | .018 |
| Caregiver-rated autonomy granting | 0.261 | 0.204 | .122 |  |  |  |
| Source | -0.267 | 8.232 | -.015 |  |  |  |
| Interaction | 0.055 | 0.384 | .064 |  |  |  |
| DV = Caregiver-rated externalizing |  |  |  | 0.92 | 3, 155 | .018 |
| Caregiver-rated autonomy granting | 0.012 | 0.197 | .006 |  |  |  |
| Source | -10.527 | 7.976 | -.595 |  |  |  |
| Interaction | 0.449 | 0.372 | .544 |  |  |  |

Note. DV = Dependent variable.
