# Supplementary material for: Individual and systemic variables associated with prolonged grief and other emotional distress in bereaved children
Source: PLoS One. 2024 Apr 30;19(4):e0302725. doi: 10.1371/journal.pone.0302725 (PMC11060573; doi:10.1371/journal.pone.0302725)
Supplement: S13 Table — (DOCX) [file pone.0302725.s013.docx]

**Supporting Information Table 13**

Regression analyses with individual and systemic variables predicting PTS functional impairment and internalizing

|  | B | SE B | β | F | DF | *R*^2^ |
| --- | --- | --- | --- | --- | --- | --- |
| DV = Children’s functional impairment linked with posttraumatic stress |  |  |  | 12.46 | 5, 152 | .30 |
| Negative cognitions | 0.059 | 0.020 | .349** |  |  |  |
| Anxious avoidance | -0.032 | 0.037 | -.085 |  |  |  |
| Depressive avoidance | 0.126 | 0.055 | .239* |  |  |  |
| Caregiver’s depression | 0.057 | 0.029 | .142* |  |  |  |
| Caregiver-rated reasoning/induction | -0.040 | 0.027 | -.105 |  |  |  |
|  |  |  |  |  |  |  |
| DV = Internalizing |  |  |  | 5.38*** | 4, 153 | .13 |
| Negative cognitions | -0.078 | 0.103 | -.097 |  |  |  |
| Anxious avoidance | 0.221 | 0.199 | .124 |  |  |  |
| Depressive avoidance | 0.594 | 0.286 | .238* |  |  |  |
| Caregiver’s anxiety | 0.469 | 0.159 | .227** |  |  |  |

Note. DV = Dependent variable. PTS = Posttraumatic stress.

* p < .05. ** p < .01. *** p < .001.
